# Evaluating and Understanding Weight Management Experiences Among Adolescent Girls During and After Residential Treatment for Obesity

**DOI:** 10.1002/osp4.70049

**Published:** 2025-03-28

**Authors:** Morgan E. Braxton, Subin Jang, Ashley M. Ruiz, Jim Hershey, Justin R. Ryder, Aaron S. Kelly, Gabriel Q. Shaibi

**Affiliations:** ^1^ Center for Health Promotion and Disease Prevention Edson College of Nursing and Health Innovation Arizona State University Phoenix Arizona USA; ^2^ Department of Pediatrics and Center for Pediatric Obesity Medicine University of Minnesota Medical School Minneapolis Minnesota USA; ^3^ Nell Hodgson Woodruff School of Nursing Emory University Atlanta Georgia USA; ^4^ GEM Academy Phoenix Arizona USA; ^5^ Ann & Robert H. Lurie Children's Hospital of Chicago Chicago Illinois USA; ^6^ Northwestern Feinberg School of Medicine Chicago Illinois USA

**Keywords:** adolescent girls, lifestyle intervention, obesity, residential treatment

## Abstract

**Background:**

Residential programs have been utilized for the treatment of adolescents with severe obesity, yet few have been evaluated.

**Objective:**

The objectives were to (1) evaluate the effect of a long‐term residential treatment program focused on treating adolescent girls with obesity and (2) explore girls' perceptions of weight management during and after participating in the program.

**Methods:**

A mixed‐methods approach was used to examine changes in weight outcomes over time among adolescent girls who completed the program (*N* = 12), and conduct qualitative interviews to explore perceptions of weight management after completion (*N* = 5).

**Results:**

Girls in the program showed a reduction in mean BMI of 16.1 ± 4.2 kg/m^2^ (−36.3% ± 5.9%) over a mean of 57 weeks. At follow‐up, three participants regained weight while two maintained their completion weight. The program shifted girls' health goals from weight loss to improved overall health. Experiences of social connection and disconnection were identified as components that impacted weight management trajectories over time.

**Conclusion:**

This program demonstrated clinically meaningful improvements in BMI. The structured nature and the emphasis on therapeutic methods were key components of the program. Social support was identified by participants as being integral to successful weight maintenance over time.

## Graphical abstract

A mixed‐methods approach was used to examine changes in weight outcomes over time among 12 adolescent girls who completed a long‐term residential treatment program, and then 5 qualitative interviews were conducted post‐completion to explore perceptions of weight management. Girls in the program had a reduction in mean BMI of 16.1 ± 4.2 kg/m2 (−36.3% ± 5.9%) over a mean of 57 weeks. The program shifted girls' health goals from weight loss to improved overall health.

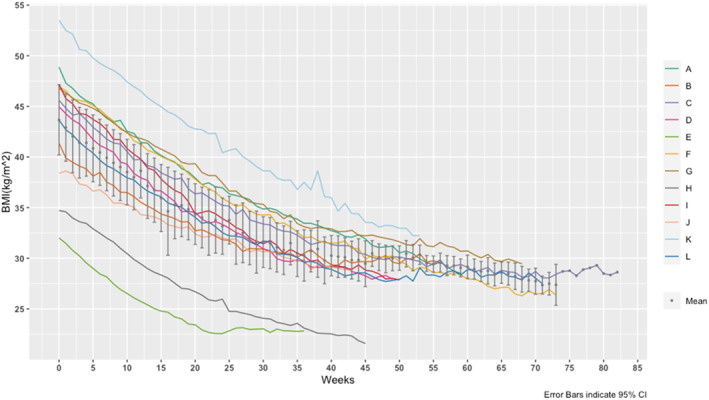

## 1 Introduction

Currently, more than 14.7 million youth are affected by obesity in the United States, nearly 20% of the population [1]. Youth with obesity are at increased risk for poor mental and physical health outcomes, including depression, type 2 diabetes, and cardiovascular disease [2]. The American Academy of Pediatrics (AAP) clinical guidelines prioritize intervention and treatment for overweight and obesity in youth [3]. Although treatment options include lifestyle intervention, pharmacotherapy, and bariatric surgery, lifestyle intervention remains the cornerstone approach for treating children with obesity [3]. However, the weight loss experienced in response to lifestyle intervention is often modest (∼1–3% body mass index (BMI) reduction) and rarely leads to clinically meaningful reductions [3].

For youth with severe obesity, residential immersion programs can offer a more intensive option for supporting weight loss compared with standard lifestyle modification. These programs can range in length (from 10 days to 10 months) but are typically short‐term, such as summer camps [4, 5, 6]. Most programs include a diet component, typically caloric restriction, and physical activity, ranging from 1 to 6 h per day, with a wide variety of activities [5]. Culinary skills, nutrition education, social support, and/or family engagement are other components commonly interwoven into these immersion programs [5]. Weight loss outcomes of residential treatment programs vary in the literature, and long‐term weight loss outcomes among youth are even more limited; some have found sustained improvements in BMI [4], while others have more variable outcomes [5, 7]. Programs that integrate cognitive‐behavioral therapy (CBT) have demonstrated greater reductions in BMI [5, 8]. The GEM (“Gratitude, Empathy, Mission”) Academy is a residential obesity treatment program based in Scottsdale, Arizona, that prioritizes adolescent girls with obesity. The program is designed to be 12 months and includes several components: academics, nutrition, fitness, multi‐modal behavioral therapy, community service, and experiential learning. The program is structured around an organized daily schedule with set sleep times, waking at 6:30 a.m., and in bed by 10:30 p.m. Girls eat three meals per day in a group setting at a family‐style table. Meals at GEM include a “controlled portion,” which is an entree between 300 and 400 kcal, as well as “uncontrolled foods,” or side dishes consisting of variously prepared vegetables, soups, salads, etc., which the students can eat ad libitum using their own hunger cues. Girls are required to track all of their nutrition intake in a food journal. Physical activity includes a daily three‐mile morning walk and an additional hour of group exercise in the afternoon, such as Zumba, resistance training, Pilates, or sports. GEM Academy (https://gemacademyaz.com/) also uses multiple therapy techniques to address the emotional and psychological aspects of weight management. Cognitive‐behavioral therapy (CBT) is the primary model used in GEM, which has been shown to demonstrate greater reductions in BMI in previous research [5, 8] Girls also participate in family, group, and individual therapy. All girls participate in multiple therapy sessions weekly in order to encourage sustainable lifestyle changes and improve their overall well‐being. Licensed therapists specialized in adolescent obesity design individualized therapeutic regimens to meet specific needs. Additional therapeutic techniques that can be used include Exposure and Response Prevention therapy (ERP), Dialectical Behavior Therapy (DBT), and Eye Movement Desensitization and Reprocessing (EMDR) [9]. All modalities used correspond to needs expressed through the reported lived experience of the participants. These additional forms of therapy help girls process traumas they may have experienced while growing up, and provide a safe and healthy space to process and respond, in order to promote having a healthy transition back home [9]. For academics, girls follow a homeschool program, meeting in the on‐site classroom for their coursework. Girls enrolled in the program also participated in community volunteer work and experiential learning opportunities. Experiential learning includes going to a public gym, bathing suit shopping, and running a 5K. The goal of these learning opportunities is for girls to learn how to adjust to regular activities outside of GEM.

The purpose of this study was to (1) evaluate the impact of a long‐term residential treatment program focused on treating adolescent girls with obesity and (2) explore girls' perceptions of weight management during and after participating in the program.

## 2 Materials and Methods

### 2.1 Study Design

A mixed‐methods approach was utilized to quantitatively examine changes in weight outcomes over time and qualitatively explore perceptions of weight management after graduating from GEM Academy, a residential obesity treatment program. Initial height was reported, as well as weekly weight, which was measured by program staff, and self‐reported height and weight at follow‐up. Qualitative interviews were guided by Pickett et al.‘s middle range theory of weight management [10], which was used to consider weight control within the wider concept of health among a subset of girls who completed the residential program. The interviews focused on contextual factors, weight management agency (knowledge, motivation, beliefs), and behaviors (physical activity, eating behaviors) that ultimately influence weight control over time [10]. The weight and height data from the GEM program were analyzed retrospectively, while participants provided informed consent for qualitative interviews and self‐reported current weight under a protocol approved by the Arizona State University Institutional Review Board.

### 2.2 Sample and Setting

Participants were eligible to participate in this study if they graduated from a residential obesity treatment program based in Scottsdale, Arizona, called GEM Academy LLC. A total of 12 adolescent girls (14–18 years) had successfully graduated from the GEM Academy and were therefore eligible to participate. Weight and height data from these 12 adolescent girls were available for analysis. Of the 12, five participants agreed to participate in qualitative interviews; the other seven did not respond to recruitment efforts or declined to participate.

### 2.3 Data Collection

Initial height and weekly weight from baseline to graduation in 12 residents were deidentified and securely shared with the research team for analysis. These 12 girls were recruited to participate in follow‐up interviews after graduation. Of the 12, five participants agreed to participate in qualitative interviews; the other seven either did not respond to recruitment efforts or declined to participate. The five girls who completed the qualitative interviews also provided their current weight at the end of their interview, which was used to examine the long‐term weight trajectory post‐graduation. Interviews were conducted over a virtual platform and audio recorded. The interview guide consisted of semi‐structured interview questions, broadly inquiring about participants' experiences in the program and transition after graduation. Audio recordings of interviews were transcribed by an independent service and checked for accuracy by the interviewer.

### 2.4 Data Analyses

Quantitative data were summarized using frequencies and percentages for categorical variables and means and standard deviations for continuous variables. Outcomes of interest were BMI, % Change in BMI from baseline, and categorical change in weight status using BMI cutpoints. BMI percentile was calculated using age‐ and sex‐specific growth charts from the CDC [11]. BMI category was defined using the BMI percentile range: normal weight (BMI percentile 5th—< 85th), overweight (BMI percentile ≥ 85th—< 95th), obesity (BMI ≥ 95th—< 120% of the 95th percentile), and severe obesity (BMI ≥ 120% of the 95th percentile). All analysis was performed using *R* version 4.2.1.

For qualitative data, reflexive thematic analysis (RTA) was used to identify salient themes [12]. RTA entails the following six steps: (1) familiarization with the data, (2) identifying initial codes, (3) generating themes, (4) reviewing potential themes, (5) naming and defining themes, and (6) developing a report of the analysis [13, 14]. RTA provides an opportunity to identify themes within the data that are informed by the theoretical assumptions of Pickett et al.‘s middle range theory of weight management, as well as the researchers' expertise in pediatric obesity. Two members of the research team independently reviewed transcripts to become familiarized with the data. Initial codes and themes were collectively identified through group discussions [15]. Potential themes, and naming and defining themes, were reviewed and refined in order to move from descriptive codes to interpretive themes [10, 12].

## 3 Results

**FIGURE 1:**
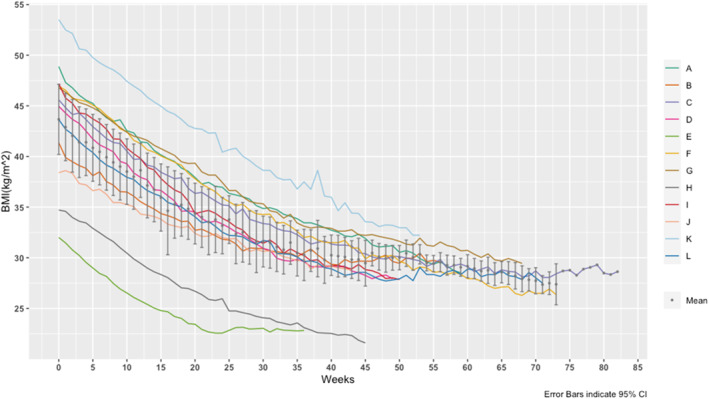
Individual BMI (kg/m^2^) changes over time. The graph depicts individual girls' (listed as A‐L) BMI weekly while enrolled in the program.

**FIGURE 2:**
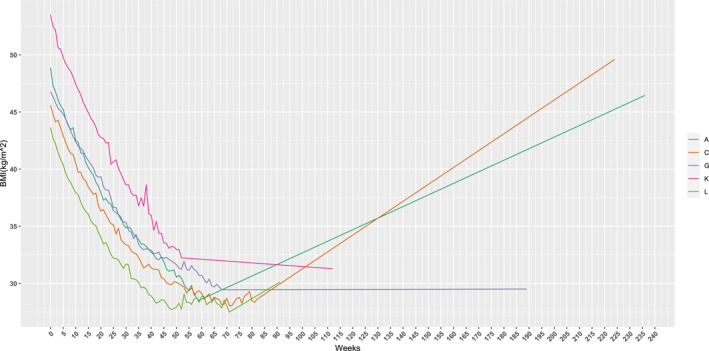
Individual BMI (kg/m^2^) trends over time: extension to the interview date data. For the five who participated in the follow‐up interviews, the graph depicts the individual girls' BMI weekly while enrolled in the program, and then at follow up.

Residents (mean age of 16 ± 1 years old) average duration in the facility was 57 weeks (range: 36–82 weeks) and mean BMI at baseline was 43.7 ± 6.1 kg/m^2^. Upon graduation, residents demonstrated a 36 ± 6% reduction in BMI from baseline, which is 16.1 ± 4.2 kg/m^2^ on average. Figure 1 depicts individual trajectories of changes in BMI over time, which demonstrates that all participants decreased their BMI while in the residential program. One participant moved from the obesity category to the normal weight category. Among the 11 participants who were initially in the severe obesity category, 6 reverted to the overweight category, 4 to the obesity category, and 1 to the normal weight category. Five residents agreed to participate in follow‐up interviews (ranging from 5 to 41 months since graduation) and provided self‐reported weight. Three participants regained some or all of their weight (Participants A, C and L), while two (Participants G and K) were able to maintain their graduation weight, although follow‐up time differed (see Figure 2).

During follow‐up interviews, participants described their experiences at GEM by outlining the elements of “the GEM Way” as well as a shift they experienced in their health goals. Experiences of social connection and disconnection were identified as factors that impacted their weight management trajectories. Lastly, participants perceived their experiences transitioning out of the residential program as “cultural shock” within a new reality.

### 3.1 The GEM Way

GEM was described as “structured,” which was usually viewed as positive by participants. Participant A said, “And just overall how regimented and scheduled it was really helped you see how much you could get done in a day…” Participant K also appreciated the structure, saying “6:30 to 10:30, whole day, planned out… It was great. Some people don't like structure like that. Me, I'm a very structured person. If someone tells me what to do and has it all planned out for me, I'm good. I can follow it to a T.” Participant L said, “And I think just the overall environment has just been very beneficial to accomplishing my goal because of the sleep schedule, the eating schedule, the exercise schedule, and just overall the support around the therapy and like the therapeutic aspects of the program.”

**Meals.** Participants commented on eating at GEM with structured meal times, portions and setting. Participant A said, “…I think for a lot of these girls, I think it was the first time family dinner and family meals were such a normal occurrence.” Three participants said they did not feel hungry while at GEM; they did not feel there was a shortage of food available and appreciated the structure of the day to always know when meal times were scheduled. One participant discussed feeling full after just the meal and mentioned food journaling as a beneficial part of the program, learning to track macros. However, Participant G described meal times as a “race to eat, so you could get the food you wanted,” referring to “uncontrolled” foods (e.g. fruit, yogurt) available after finishing your pre‐portioned meal.

**Exercise.** Exercise included a 3‐mile morning walk and a variety of alternate activities. Participant A said, “Like they weren't just sticking us on a treadmill. They were making it enjoyable. They wanted us to see that exercise could be a sustainable habit that you could enjoy. Not something, you had to do. Um so, I really appreciated the variety in which they made exercise accessible to us.” Participant L referred to exercise as “therapeutic” and described her mental health improving as a result of exercise.

**Therapy.** Girls discussed the therapeutic aspects as an important part of their weight loss journey. Participant L stated, “Because I feel like being obese has its trauma because a lot of people look at you because the beauty standard is just not it sometimes. And I think that therapy really helps unravel that and understand it. And help you learn that it is not your fault. You know, like we can take accountability for some things, but there's some things we can't control and those things that we can't control are the things we blame ourselves for.”

**Sleep.** While sleep schedules outside of GEM were described as a challenge, girls spoke very positively of the regulated sleep schedule they maintained in GEM. “Like I didn't even worry about anything. I was just like I want to go to bed. Good night. Loved it” (Participant L). Participant K said, “I go to bed exhausted after that long, long day I had filled with activities, get like the best night sleep ever, eight hours too; man, I'm telling you, never had it.”

**Volunteering.** Girls spoke positively about the service component of the program, enjoying the time and variety of ways they were able to serve their community. Participant L explained the value of service work to her, “And like it just gives you purpose, you know, when you don't think you have purpose. And I think that's a huge part of GEM that I really enjoyed. It was definitely the volunteer outings because it just, it was so fun and it was like a way for us to learn that we could help people without giving too much of our lives, you know.”

**Experiential learning.** “[They] made events that would push our boundaries because we did not like to do certain things, but [they] knew that we needed to break out of that shell especially if we were going to go back home to set environments, like if we didn't like to do something at home, we're going to have to learn how to do it there because it's better to learn in a safe, protective environment…” (Participant K). These experiences included participating in fun run races and shopping for bathing suits.

#### 3.1.1 Goal Shift

Participants described a transition of their goals over time, from “losing weight” or “being skinny” to a goal of attaining mental and physical health. Participant L said, “My goal going in was to lose weight because I felt like I was being forced to lose weight. But then my goal actually changed to be the healthiest I could be.” New goals surrounding health included becoming physically fit, trying out for a sports team, or being able to play with younger siblings. Participant A talked about this goal transition, saying, “At the time, you really just want to be liked, by boys, by people; you want to be equal. You want to be seen. And I think that was a lot of the motivation moving toward I just want to be skinny and pretty and I want boys to like me. Now those goals are very different. You know, I want sustainable endurance; I want to be healthy for my future kids. I want a lot more sustainable and real reasons to take care of myself and why I keep popping back on the horse when things are not as you originally planned. That was quite the process at GEM to re‐evaluate my goals, which they are really good about helping you really sit down and decide healthier and sustainable goals that aren't just surface level and shallow.”

### 3.2 Experiences of Social Connection and Disconnection

All participants discussed the impact that social connections and disconnections inside and outside the program had on their trajectories in managing weight. GEM was described by 2 girls as a “bubble.” Within the program, the girls were a source of social support for one another. Activities at GEM have fostered experiences for establishing new experiences for social connection. For example, Participant C mentioned that her friends back home “were all smaller and they didn't understand [what it was like to be obese],” but while at GEM, “…you're trying new things. Like uh, things you've never done before that you never… you don't want to do like on purpose just being heavier…going out to events and dressing up, like going to the gym, things like that were difficult being heavier [so] on purpose that you would avoid [them]…”

All participants discussed experiences of social disconnection, including losing friendships before starting the program, having friends and family who did not understand, and challenges in relationships with family members as they progressed in the program. For example, Participant A described the home visit, saying, “… There are just a lot of growing pains that come along visiting home when you are no longer the person that fits into the dynamic you've always had with your parents or your siblings or your friends…You think it's going to be just fun and games. Until you realize that you are not the person that you were there and everyone else has to kind of figure it out how they interact with you.” Often, returning home included navigating challenging situations, particularly within the family. Participant C said, “I think that's my biggest trigger, just being around my family.” Participant L said, “…Just like the overall stress in the family. Like, uh, the dysfunction before GEM was still there after GEM…like things don't necessarily change between you and them. Like you've changed, right, and you're able to go about a situation a lot more healthy. But like we still want to bite back onto that hook, you know.”

After leaving the program, all girls discussed how social connections shaped their ongoing relationship around eating and managing weight. For example, Participant G, who maintained her weight loss after leaving GEM, said, “Especially my dad is like my biggest support system. Like he helped me a lot coming home with cooking and meal prepping and helping me stay on track with everything.” Parents, aunts, and grandparents were mentioned as support people in weight maintenance for participants when they transitioned home. However, sometimes, support people were not helpful. Participant C, who regained weight after leaving GEM, said, “…My boyfriend… I just think we both struggle with food and so it also affects us because we've built this bond around eating. And so, breaking that, we're gonna have to mourn our whole relationship.”

### 3.3 Cultural Shock Within a New Reality

All girls discussed the difficulty of transitioning home following GEM. Participant K said, “But I was excited to go home but I guess I just wasn't prepared to go home. Because when I went home it was just a cultural shock.” Cultural shock within a new reality entailed phone boundaries, the initial transition process, and adjusting to self‐initiated habits.

#### 3.3.1 Phone Boundaries

During GEM, girls discussed missing their phones at first, describing it as a shock and missing it as a means to contact people and a distraction when they felt they needed one. Upon graduation, most participants discussed regaining access to technology and a heavy amount of time being on their phone “making up” for all the things that they missed. Participant C said, “Because I was so focused on like having my phone back and having the TV there and all the shows that I missed and all these things that I wanna watch now. I had Instagram, there's TikTok and like, oh my God, I was just on my phone. I was just on my phone 24/7.” While most girls found themselves putting excessive time into being on their phone, Participant G thought having her phone back was a positive; it was a way to talk to friends while going on walks, and she felt she could set her own boundaries with her phone use. Participant A discussed the benefits of GEM limiting phone access, saying, “As you grow older and you look back the reason GEM was so effective is because you weren't just getting that constant stimuli of that outside world, of expectations, of beauty standards, of everything in between and you really just had to sit down and it forced you to be present with yourself, which I think is a huge part.”

#### 3.3.2 Transitioning Home

The process of transitioning home was iterated as a cultural shift that encompassed both a “honeymoon bliss” to being done with the program but also “terrifying” and “isolating”.” Upon returning home, girls described behaviors, either a hyper‐focus on eating or a fear of gaining weight. For example, Participant K described her propensity toward overeating, saying, “So within that period I was like binging on anything and everything I could get my hands on because I felt like I missed out on it or like I was never going to have it again…I was ravenous for it. I was like, every time I saw something that I want I didn't stop myself from getting it. I absolutely did not. I was like, ‘Because I'm home now and because no one can tell me otherwise I'm going to go get it.’” Meanwhile, Participant G described her tendency toward restrictive eating upon coming home, “I was so scared when I got home that I was going to gain weight, so my first like response was just like not to eat and so that was personally, I know something I struggled with was like when I got home. I lost more weight, but it wasn't in a healthy or good way because I was just so scared. Even if I was hungry, I would just like chew gum, chew ice cubes, and drink water. Like I was just so scared to eat. And so that's one thing where I know I definitely struggled with was like doing it to an extreme.”

#### 3.3.3 Self‐Initiated Habits

As participants re‐entered normal life, they had to establish their own health patterns outside of GEM. For example, Participant A said, “I think it's just transitionary being in your 20s thing that before it was like, okay, GEM bought the groceries, they set out the recipe and all the food for you and you cooked it and then you would eat it. And now it's almost in that environment where it's like, it's on you now. So that's been a growing pain that I forgot. It would have been easier if I had kept it established.” Participant L discussed her transition to home life saying, “…Tracking the food just kind of felt like a hassle to me. Like I just, I just didn't want to do it honestly. Um, and the exercise also felt like a hassle, even though I know it feels good to me to exercise. It was just like I would rather do this than do that, you know. Like I'd rather hang out with my friend rather than, you know, um do some dancing videos, you know. So, it's like that feeling of priorities, that's kind of changed a little bit more because like more things entered my life. Like more friends, new friends entered my life, you know. More hours at school, more hours of homework. Like these things just kind of entered and things just left.”

Some participants mentioned therapy, exercising, and mindful eating as patterns they maintained from GEM. Tracking food by hand in a notebook was not a continued health practice after leaving GEM for any of the participants, although some mentioned they did track their food in an app. Sleep was something that participants knew was important but struggled to maintain. Participant A described her process in maintaining her GEM habits over time, “I mean stress exists through GEM but so do the tools and sometimes it's harder to remember you have the tools outside of GEM. Um, so I mean the past couple of years I've had breakups, I've had um…college changes, career changes, I've moved a couple times. I've moved in with people, I've moved out on my own. Um, Life happened and um…I mean I think I'm still on the verge of really establishing those habits as the normal. I think I'm just slowly checking off boxes. Okay, that's the habit again. It's happening, it's happening; it's just like consistently going to the gym every day. The next one is like doing my morning walks. There's that but also I need to get better at meal prep.” The added stress of “normal life” made it challenging to maintain the health practices learned in the program.

## 4 Discussion

The GEM program resulted in substantial decreases in weight measures among the 12 graduates [16]. The regimented schedule and emphasis on mental health were key components of the program that participants cited as being integral to their success. Of the five girls who agreed to participate in the qualitative interviews, two maintained their weight loss over time, and three experienced weight regain. Reasoning for this weight regain is a complex intersection of factors, including biology, behavior, and environment [17].

All participants discussed a shift in their health goals over time, from an emphasis solely on achieving weight loss to a desire to sustain overall health, which included managing their mental health. Similarly, past research on short‐term weight loss programs has demonstrated significant improvements not only in weight‐specific outcomes but also in self‐worth, regardless of the amount of weight lost, suggesting the positive psychosocial impact of a group weight loss program and of creating social connections with similar individuals [18]. Other research in short‐term programs found decreases in depressive symptoms and negative thoughts, and improvements in quality of life were correlated with weight loss [19, 20, 21].

Girls discussed experiencing either a hyper‐focus on weight maintenance or an uncontrolled “honeymoon” period of overeating upon returning home. Additionally, in the long term, they reported mixed results on the health habits they either were or were not maintaining after transitioning home. Sustained weight loss is a challenge; there are multiple factors, including biology, behavior, and environment, that collectively promote weight regain [17]. This is why a long‐term strategy, including support and weight‐maintenance‐specific counseling are needed to support individual's weight loss goals [17], as well as consideration of pharmacotherapy and bariatric surgery when appropriate [22]. Closer clinical monitoring and support during this initial transition period and beyond may benefit future graduates by promoting healthy lifestyle habits and long‐term weight regulation [17].

These data demonstrated the perceived importance of social relationships, particularly family engagement, as key to weight maintenance, which is in line with previous research [23, 24]. Even more importantly, in the transition home, all girls talked about the people in their lives that supported their health behaviors or were perceived as barriers to maintaining their goals. Social connection impacts health outcomes, and with the high rates of loneliness in the United States [25], our results suggest that it is important for girls transitioning out of intensive programs to have a well‐established network of support people in their lives to increase the likelihood of success. Furthermore, many of these participants were transitioning home during the COVID‐19 pandemic, which was associated with decreased social interaction and increased loneliness [26]. This added unique stress and challenges to engage in healthy relationships could have potentially contributed to the weight regain of some participants after graduating.

Participants reported an almost obsessive nature of interacting with their cell phones upon leaving the program and having access to them again. The girls who participated in this program are part of a generation of “digital natives,” having grown up with the internet always available [27]. This group is accustomed to almost continuous access to technology and social media, with access to smartphones [28]. Adolescents and young adults report high levels of attachment to their devices, and there are both benefits and harms to individuals [25, 28]. In part, cell phones can foster connection through online platforms, which provide information and social support [25]. However, there can be struggles with cell phones dominating attention, therefore minimizing in‐person interactions, and can even lead to greater levels of loneliness [25]. In addition, smartphone dependency is predictive of loneliness and depressive symptoms [29]. The previous data on smartphone use aligned with the findings in this study, which showed that cell phone use did impact participants' health goals, both positively and negatively. Most participants cited this as a huge time constraint, a need to “catch up” on all the media previously missed, and being on their phones “24/7”. However, one participant cited the benefits of using her phone to maintain social connection and health goals at the same time (e.g. going on a walk while talking on the phone to a friend).

A major challenge that participants cited was the struggle with the transition out of the GEM Academy program, feeling like it was harder than they had anticipated and not feeling prepared. Challenges to maintaining health gains were heavily laced in social support and added stress of reentering “normal” life, including added daily stressors (e.g., school, work, relationships, cell phone access), making it difficult to prioritize health‐promoting behaviors. Understanding exactly why some participants regained weight while others maintained their weight loss is complex. However, among the girls who did maintain their weight loss over time (Participants G and K), both discussed a strong support system, while those who experienced regain (Participants A, C, and L) discussed challenges with social support. Again, the COVID‐19 pandemic potentially played a role in this for some participants, as social isolation was a common experience [26].

This study provides novel qualitative and quantitative data on a residential obesity treatment program in which adolescent girls with severe obesity experience significant weight loss. The qualitative analysis provides an additional nuance of the participants' views on the program and highlights the social support needed for success. The follow‐up data demonstrate the challenging nature of long‐term weight maintenance after transitioning out of care. However, this study is limited by the small sample size, retrospective design, the lack of a comparison group, and potential for selection bias in those who chose to participate in the follow‐up. Also, it is a limitation that follow‐up weight data were self‐reported, as it is not possible to verify accuracy. Future research could follow girls through their time in and after the program, assessing biological, behavioral, and environmental factors in order to better understand how these factors impact outcomes.

## 5 Conclusion

This study assessed the short‐ and long‐term effects of a residential weight loss program and demonstrated clinically meaningful improvements in BMI. The structured nature and the emphasis on therapeutic methods were identified as key components of the program. Social support was integral to the success of girls who maintained their weight over time. Future studies are needed to consider the long‐term implications of participating in this type of residential weight loss management program.

## Author Contributions

All authors participated in conception of this work. M.E.B. conducted qualitative interviews. M.E.B. and A.M.R. analyzed qualitative data. S.J. conducted quantitative data analysis. All authors were involved in writing the paper and had final approval of the submitted and published versions.

## Conflicts of Interest

Dr. Kelly engages in unpaid consulting and educational activities, as well as serves as an unpaid investigator for Novo Nordisk; engages in unpaid consulting activities and serves as an unpaid investigator for Boehringer Ingelheim, Eli Lilly, and Vivus; and receives donated drug/placebo from Novo Nordisk and Vivus for National Institute of Diabetes and Digestive and Kidney Diseases (NIDDK)‐funded clinical trials.
